# Attitudes and Knowledge Regarding Autism in Israeli Society

**DOI:** 10.1177/23969415261454218

**Published:** 2026-06-08

**Authors:** Sara Ferman, Osnat Segal

**Affiliations:** 1 ¹Department of Communication Disorders, Faculty of Medicine, Tel Aviv University, Israel; 2 Ono Academic College, Kiryat Ono, Israel; 3 Sagol School of Neuroscience, Tel Aviv University, Israel

**Keywords:** autism, attitudes, knowledge, neurodiversity, cultural diversity

## Abstract

This study examined societal attitudes and knowledge regarding autism within Israel's culturally and socioeconomically diverse population. A total of 2,377 respondents completed the *Societal Attitudes Towards Autism* (SATA) questionnaire, translated into Hebrew and Arabic. The survey examined the effect of socio-demographic variables, including gender, age, education, income, ethnicity, and familiarity with autism or knowledge about and attitudes towards autism. Overall, respondents demonstrated positive attitudes (*M* = 69.5%) and relatively high levels of knowledge (*M* = 64.6%) about autism. Most respondents reported positive (56%) or very positive (35%) attitudes towards autism, and 91% of the participants expressed openness and acceptance towards autistic individuals. A substantial proportion of the respondents reported a high (56%) or very high (24%) level of knowledge about autism, suggesting that 80% the Israeli public is relatively well-informed. Women, midlife (30s to 50s) adults, respondents with higher education and high income, and respondents who were acquainted with an autistic individual reported more positive attitudes and had significantly greater knowledge about autism. Levels of autism acceptance and knowledge varied across ethnic groups, with Jewish respondents reporting more positive attitudes and greater autism-related knowledge than Muslim, Druze, and Christian respondents. Hierarchical regression analyses revealed that the level of knowledge was the strongest and most consistent predictor of positive attitudes. The findings underscore the importance of educational, socioeconomic, and cultural influences on public perceptions of autism. Promoting autism acceptance in multicultural societies such as Israel requires knowledge-based and culturally responsive educational programs, alongside opportunities for meaningful social contact between autistic and non-autistic individuals.

## Introduction

Autism is a lifelong neurodevelopmental condition that shapes how individuals perceive, interpret, and engage with their surroundings. Recognized today as part of the natural spectrum of human neurodiversity (Bottema-Beutel et al., 2021), autism is characterized by two core domains: (a) persistent difficulties in social communication and interaction, and (b) restricted and repetitive patterns of behavior, interests, or activities (American Psychiatric Association, APA, 2013). While many autistic individuals face challenges in achieving full independence, numerous adults actively engage in employment, community life, and self-care (Iwasa et al., 2022).

Over recent decades, reports of autism have become considerably more frequent across the globe (Shaw et al., 2023; Talantseva et al., 2023). In Israel, longitudinal data comprising 3.5 million births from 2000 to 2020 show nearly twice as many diagnosed cases among children aged 1–17 compared to earlier years. The most notable changes were observed among the youngest cohorts, including 2–3-year-olds (from 0.27% to 1.19%) and 4–6-year-olds (from 0.8% to 1.83%), pointing to both broader recognition and earlier detection (Dinstein et al., 2024). These trends underscore that autism is common, and most people are likely to encounter autistic individuals in their personal, educational, and professional lives (Dillenburger et al., 2015; Tipton & Blacher, 2014).

In parallel with increasing rates of diagnosis, questions remain about how well the public understands and accepts autism. Addressing this issue is essential within the framework of the neurodiversity movement, which challenges deficit-oriented perspectives and emphasizes acceptance, inclusion, and appreciation of diverse neurological profiles (Ortega, 2009). Evaluating public perceptions is, therefore, a crucial step towards fostering acceptance, reducing misconceptions, and promoting a more inclusive view of autism as a natural expression of human diversity.

### Knowledge and Attitudes About Autistic People

Attitude is a learned tendency that shapes how individuals think, feel, and behave towards people, objects, and situations in their environment (Eagly & Chaiken, 1993). It reflects a person's predisposition to respond positively or negatively to social stimuli and is influenced by cultural norms; behaviors regarded as typical or acceptable in one society may be perceived differently in another culture (Eagly & Chaiken, 1993). By comparison, knowledge refers to information, facts, and skills acquired through experience, education, and interaction with the environment (Jorm, 2000). Whereas attitudes determine how individuals feel and behave, knowledge provides the conceptual foundation that informs those attitudes and guides decision-making (Schrader & Lawless, 2004).

A growing body of research shows that societal knowledge and attitudes towards autism profoundly shape the lived experiences of autistic individuals. Positive or negative perceptions within a community can influence opportunities for social inclusion and acceptance (Gillespie-Lynch et al., 2015; Sasson et al., 2017), affect mental health and self-perception (Cooper et al., 2017; Crane et al., 2019), and determine the extent to which autistic people are supported in educational, healthcare, and occupational contexts (Crane et al., 2018; Scott et al., 2017; Segall & Campbell, 2014). Family well-being and the development of self-advocacy and identity are also closely tied to societal understanding and attitudes (Botha & Frost, 2020; Burrell et al., 2017; Westbrook et al., 1993). Collectively, these findings highlight how societies perceive autism—through both knowledge and their affective stance.

There is growing evidence that demographic factors such as gender, age, education, income, ethnicity, and personal connections to individuals with autism can influence public knowledge and attitudes towards autism. Developing a systematic understanding of how these factors shape awareness and perceptions is essential for guiding public education efforts, reducing stigma, and fostering more inclusive and accepting communities.

### Gender

Gender may play a role in shaping attitudes and knowledge about autism (Kuzminski et al., 2019). Several studies indicate that women tend to hold more positive attitudes and demonstrate higher levels of autism-related knowledge than men (Durmaz et al., 2025; Karacan Doğan et al., 2023; Kuzminski et al., 2019). A study conducted in Turkey found that female students demonstrated significantly higher autism knowledge scores than male students, while no gender differences were found in overall attitudes or comfort levels (Karacan Doğan et al., 2023). Additional research reports that women tend to display a greater understanding and more favorable attitudes towards autism than men in Saudi Arabia (Alsehemi et al., 2017), Ireland (Dillenburger et al., 2013), and the United States (Tipton & Blacher, 2014). Collectively, these findings suggest that gender may influence both the depth of knowledge about autism and attitudes towards autism, with women often exhibiting more positive and informed perspectives.

### Age

Research on the influence of age on attitudes towards autism reveals complex and sometimes contradictory findings. Some studies indicate that older adults may hold more negative views towards individuals with mental illness, possibly reflecting generational differences in exposure to mental health education or prevailing social norms during their upbringing (Schomerus et al., 2015). For instance, older respondents in this study tended to express greater social distance from people with psychiatric conditions. In contrast, other research has shown that older adults may display more positive help-seeking attitudes, which means that they are more open to consulting professionals for psychological difficulties. This reflects an increased awareness of mental health care and greater trust in medical systems (Alsehemi et al., 2017; Mackenzie et al., 2006).

When focusing specifically on autism, however, studies have found no consistent relationship between age and attitudes (Shand et al., 2020). Instead, contextual factors, such as education, personal experience, and direct personal contact with autistic individuals, have emerged as stronger predictors (Kuzminski et al., 2019; Shand et al., 2020).

### Education and Income

Socioeconomic status (SES) has been increasingly examined as a factor influencing public attitudes and knowledge towards autism. While income appears to contribute to these differences, its influence is often intertwined with educational attainment and exposure to autism.

Higher levels of education and income are consistently associated with greater knowledge and more positive attitudes towards autism (Alyami et al., 2022; Dillenburger et al., 2013; Kouznetsov & Jelastopulu, 2023). Research indicates that university students, particularly those studying disciplines such as psychology and health sciences, tend to hold fewer misconceptions and display more favorable attitudes towards inclusion and interaction with autistic individuals (Gardiner & Iarocci, 2014; Nevill & White, 2011; Zarokanellou et al., 2025). Moreover, targeted autism education and awareness training have been shown to enhance understanding and reduce stigma, especially among individuals with initially limited knowledge (Gillespie-Lynch et al., 2015).

Al-Juhaishi et al. (2025) reported that caregivers in Iraq with low family income demonstrated reduced autism knowledge and more negative attitudes. However, this effect became insignificant once education was controlled. In a large-scale Lebanese survey, Younes et al. (2025) similarly found that higher autism knowledge was associated with lower stigma, but income was not an independent predictor once prior awareness was included in the model. Studies conducted in Malaysia and Saudi Arabia further support this pattern, showing that individuals with high income and education levels tend to exhibit greater awareness and more favorable attitudes towards autistic individuals (Al-Ruwaili et al., 2024; Kaman et al., 2023). Comparable trends have been documented in Western contexts, in which socioeconomic disparities shape opportunities for early diagnosis and access to accurate information. For example, Thomas et al. (2012) found that children from high-income families in the United States were more likely to receive an autism diagnosis at an earlier age, reflecting greater parental awareness and resource availability. Likewise, Cage et al. (2018) observed that higher educational attainment in the United Kingdom was linked to more accepting attitudes towards autistic individuals.

Collectively, these findings suggest that although high income may correspond with enhanced understanding and acceptance of autism, its effects are largely mediated by education and personal experience, underscoring the importance of educational interventions and public awareness initiatives across all socioeconomic groups.

### Culture and Ethnicity

Culture, which is defined as the shared values, beliefs, and customs transmitted across generations, plays a central role in shaping individuals’ attitudes, emotions, and behaviors through social norms, institutions, and collective belief systems (Jorm, 2000; Markus et al., 1996). Cultural frameworks influence how people perceive and respond to disability and neurodiversity, leading to variation in public attitudes towards autism across societies. Studies from Saudi Arabia (Alyami et al., 2022) and Malaysia (Azmi et al., 2022) highlight persistent gaps in knowledge and widespread misconceptions about the causes and treatment of autism, reflecting culturally rooted understandings of disability.

Empirical evidence further indicates that cultural and social context shape not only attitudes towards autism but also their variability across populations. A meta-analysis of 16 studies on mainstream teachers’ attitudes towards children with autism revealed a broad range of perspectives—some positive, others neutral, and a few negative—suggesting that attitudes are influenced by both personal and contextual factors (Gomez-Mari, Sanz-Cervera, & Tarraga-Minguez, 2022; Kuzminski et al., 2019). Despite these differences, research points to the persistence of negative stereotypes, including the dehumanization and avoidance of autistic adults (Cage et al., 2018; Kofidou & Mantzikos, 2020). Moreover, general knowledge about autism remains uneven and limited in many regions. Studies in Lebanon (Rouphael et al., 2023), the United States (Flood et al., 2013; Golson et al., 2022), Turkey (Rakap et al., 2020), and India (Barik et al., 2024) consistently report low-to-moderate levels of understanding. Cross-cultural research similarly reveals variations in both knowledge and attitudes among Black, Asian, and White ethnic groups (Gemegah et al., 2021). Together, these findings underscore that attitudes towards autism are shaped by cultural experience and are strongly influenced by knowledge (Gemegah et al., 2021; Kuzminski et al., 2019).

In Israel, where the population includes approximately 74% Jews, 21% Arabs (comprising Muslims, Christians, and Druze), and about 5% other groups (Central Bureau of Statistics [CBS], 2023), cultural and ethnic diversity may similarly influence perspectives on disability and autism. Although research directly examining ethnic differences in autism-related attitudes within Israel remains limited, earlier studies have shown that cultural background can affect perceptions of disability, family response, and help-seeking behavior (Kandel et al., 2004; Weinberg & Sebian, 1980). Understanding these cultural and ethnic dimensions is essential for developing effective public education and community-based interventions that promote the inclusion and acceptance of autistic individuals across diverse cultural groups.

### Religiosity

Israeli society includes several religious sectors: *Haredi* (to be referred to here as ultra-Orthodox), Modern Orthodox (known in Israel as *Religious Zionists*), traditional, and secular Jews. The ultra-Orthodox population constitutes approximately 13% of the Israeli population and is characterized by a conservative religious lifestyle that emphasizes strict observance of Jewish law and a relatively insular community framework. *Modern Orthodox* (known in Israel as *Religious Zionists*) refers to a stream of Judaism that combines traditional religious observance with active engagement in modern society, including support for secular education, professional life, and—in Israel—a religious commitment to the Zionist project and the State of Israel (Sagi, 2023). The term *Traditional Jews* (often called *Masorti* in Hebrew, especially in Israel) refers to individuals who maintain many religious customs and cultural practices, but do not identify as fully religious or strictly observant and thus represent a range of traditional lifestyles that fall between secular and Orthodox categories (Goldberg, 2013).

Several studies suggest that stigma towards disabilities within the ultra-Orthodox community may be associated with limited exposure to and a lack of knowledge about individuals with disabilities (Freund & Band-Winterstein, 2013; Milstein & Gershoni, 2018; Shoker, 2020). Research has also indicated that families sometimes choose to conceal a child's disability due to concerns about stigma and social labeling, a phenomenon that appears more common in the ultra-Orthodox sector than in the general population (Milstein & Gershoni, 2018). In addition, stigma related to mental illness may be particularly significant in this community because of its potential implications for the family's reputation and future matchmaking prospects (Struch et al., 2007).

At the same time, the ultra-Orthodox community demonstrates a dual approach towards individuals with disabilities, namely, strong communal responsibility and support for vulnerable members alongside persistent social stigma. Nevertheless, several studies point to gradual positive changes in attitudes towards people with disabilities within the community in recent years (Lifshitz & Globman, 2006; Maman, 2014; Tzur, 2014).

The Modern Orthodox sector, which comprises about 10% of the population, combines adherence to Jewish law and tradition with support for Zionism and active participation in Israeli society. Although there are diverse responses to modernity within this group, members share common educational and social frameworks, such as participation in the state religious education system, national service programs (civilian alternatives to military service), *hesder yeshivot* (institutions combining advanced religious study with military service), and pre-military preparatory programs (called *mechinot* in Hebrew) that integrate religious, educational, and leadership training prior to enlistment in the army (Huri & Zeira, 2017).

The secular sector, representing about 43% of the population, is generally associated with secularization in which religion has shifted from the public sphere to the private domain, and social institutions increasingly operate according to independent values and norms rather than religious authority (Govni, 2014).

Research addressing attitudes across these sectors is relatively limited, yet differences are expected due to distinct educational systems, community structures, and socialization processes. For example, Ashual (2011) found that, compared with women from the ultra-Orthodox population, secular and Modern Orthodox women expressed more favorable attitudes towards both marriage to a man who stutters and his employment prospects. In contrast, Halevi-Keren (2007) reported that compared with ultra-Orthodox families, secular families tend to experience greater disruption in family functioning when raising a child with special needs.

### Knowing a Person With Autism

Research has consistently demonstrated that knowing someone with autism has a positive influence on both the level of knowledge about autism and attitudes towards autistic individuals. This is rooted in Contact Theory (Allport, 1954), which posits that interpersonal contact can reduce prejudice.

Various studies have demonstrated that people who have personal relationships with autistic individuals demonstrate a greater understanding of the diversity of the spectrum, hold fewer stereotypes and more accepting attitudes, and are more likely to support inclusion and accommodations (Morris et al., 2004; Nevill & White, 2011). For example, Nevill & White (2011) found that college students who knew someone with autism expressed significantly more favorable attitudes. However, the quality and frequency of contact are significant; superficial acquaintances may not lead to a meaningful attitude change (Campbell & Barger, 2011).

### The Present Study

Given Israel's cultural and socioeconomic diversity, examining public attitudes and knowledge about autism is particularly important. The country's varied demographic composition offers a valuable context for understanding how cultural, educational, and economic factors shape perceptions of autism. Despite increasing awareness, notable gaps remain across communities, influencing early diagnosis, acceptance, and inclusion. Recognizing these differences can inform culturally tailored interventions, improve support systems, and guide the development of inclusive policies. Ultimately, research focusing on Israel contributes to the global understanding of how cultural and demographic contexts shape attitudes towards neurodiversity.

## Method

### Participants

This study sought to recruit a sample as representative of the general population in Israel as possible, considering key demographic and cultural factors. The final sample included 2,375 individuals who completed the questionnaire in full. To correct for discrepancies between the initial sample and the general population (e.g., an overrepresentation of women and younger adults), post-stratification survey weights were applied. The weights were calculated based on the intersection of gender, age, and ethnicity (Jewish, Muslim, Christian, and Druze) using demographic data from the 2024 Statistical Abstract of Israel published by the Central Bureau of Statistics (CBS). As shown in Table 1, which presents both the unweighted and weighted distributions, the weighted sample successfully aligns with national demographic benchmarks. Participants were recruited from across Israel (northern, central, and southern regions) through WhatsApp groups and other social media platforms and represented a wide range of occupations, including engineers, teachers, and speech-language pathologists, among others. In addition, participants reported whether they personally knew someone with autism.

**Table 1.** Distribution of the Study Population Across Demographic Variables.

		Unweighted Sample		Weighted Sample	
Demographics	Category	n	%	n	%
Gender	Men	619	26	1,173	49
	Women	1,756	74	1,202	51
Age	18–29	743	31	643	27
	30–39	636	27	465	20
	40–49	495	21	423	18
	50–59	317	13	339	14
	≥60	184	8	504	21
Education	High school and below	891	38	945	40
	BA/Vocational education	855	36	809	34
	MA/PhD	628	26	620	26
Income (monthly, NIS)	Low (<10,000)	982	41	854	36
	Average (10,000–20,000)	918	39	968	41
	High (>20,000)	475	20	553	23
Ethnicity	Jewish	1,644	69	1,846	78
	Muslim	593	25	432	18
	Christian	21	1.0	55	2
	Druze	117	5.0	43	2
Religiosity	Ultra-Orthodox	436	26	468	25
	National-Religious	291	17	301	16
	Traditional	333	20	382	20
	Secular	612	37	734	39
Knowing a person with autism	Yes	1,152	49	1,145	49
	No	1,200	51	1,185	51

*Note*. Percentages are rounded to the nearest whole number unless otherwise indicated. NIS = New Israeli Shekels.

### Research Materials

For this study, the Societal Attitudes Towards Autism (SATA) questionnaire was employed. The SATA was originally developed by Flood et al. (2013) and later adapted by an Australian research group (Kuzminski et al., 2019). The present study used the Australian version translated into Hebrew and Arabic. Several items from the original English questionnaire were adapted to better reflect the Israeli context, while others were reworded for clarity. Details of these modifications are provided in Appendix A.

Permission to translate and use the SATA in both Hebrew and Arabic was obtained from the original author (Flood et al., 2013) and from the *Journal of Research in Special Educational Needs*. Additional permission to use the Australian adaptation was approved by *PLOS ONE*. To ensure accuracy, the translation process included both direct translation and back translation.

The questionnaire comprised three main sections. The first section collected socio-demographic information, including the participants’ gender, age, education level, income, religion, ethnicity, region of residence, occupation, and whether or not they knew a person with autism. The second section contained 17 statements designed to assess participants’ attitudes towards autism (e.g., “People with autism should not be in romantic relationships”). The third section included 20 statements evaluating the participants’ knowledge about autism (e.g., “Autism only exists in children”). Knowledge-related items were categorized into four domains: (a) social views and perceptions, (b) the lived experience of individuals with autism, (c) defining characteristics of autism, and (d) strengths and challenges associated with autism. In both the attitudes and knowledge sections, participants rated each statement on a 4-point Likert scale ranging from 1 (“strongly agree”) to 4 (“strongly disagree”). The items assessing attitudes showed a reliability coefficient of α = 0.768, while even higher reliability was found for the items assessing knowledge (α = 0.862). Overall, these values indicate good internal consistency.

### Procedure

Following approval from the Tel Aviv University Ethics Committee # 0005949-1, the questionnaire was prepared in a digital format and adapted to both Hebrew and Arabic to address the research questions, including demographic inquiries. The online version was implemented using Google Forms, allowing participants’ responses to be automatically recorded in a digital spreadsheet.

Prior to completing the questionnaire, participants provided informed consent and were assured that their responses would remain confidential. To maximize participation, the survey was distributed via multiple channels, including online forums, social media groups (e.g., Facebook), and snowball sampling, whereby acquaintances and family members of the research team shared the questionnaire within their respective networks. Questionnaires were completed anonymously, with access to responses restricted to the researcher, thereby ensuring data confidentiality and participant privacy. Recognizing the challenge of reaching underrepresented populations, such as individuals with low educational attainment or income who are less likely to respond to digital surveys, the research team made targeted efforts to recruit such participants in person. A paper-based version of the questionnaire was provided for this group.

### Data Analysis

All statistical analyses were conducted using the Statistical Package for the Social Sciences (SPSS, Version 26; IBM, 2019). Descriptive statistics (means, standard deviations, medians, and frequencies) were calculated for all study variables, including attitudes towards autistic individuals and self-reported knowledge about autism. Internal consistency of the questionnaire scales was assessed prior to analysis (Cronbach's *α* values).

To examine the distribution of participants’ responses, Chi-square goodness-of-fit tests were performed for both attitudes and knowledge categories using the weighted sample. Group comparisons were conducted using nonparametric tests due to the non-normal distribution of the dependent variables: Mann–Whitney *U* tests were used for two-group comparisons (e.g., gender, knowing a person with autism), and Kruskal–Wallis tests with Bonferroni-adjusted pairwise comparisons were used for comparisons across three or more groups (e.g., age, education, income, ethnicity, religiosity). Effect sizes were reported using Cramér's V for Chi-square tests and Cohen's *d* for Mann–Whitney and Kruskal–Wallis tests.

Finally, a stepwise multiple regression analysis was performed to examine predictors of attitudes towards autistic individuals. Knowledge scores were entered in the first model, followed sequentially by income, gender, and education in subsequent models to assess their incremental contribution to explained variance. For all analyses, significance was set at *p* < .05, with exact *p*-values reported when appropriate.

## Results

This section presents the findings of the study on participants’ attitudes towards and knowledge about autistic individuals, followed by analyses of differences across demographic variables and predictors of attitudes. The overall mean attitude score towards autistic individuals was 69.55% (*SD* = 13.88), with a median of 68.63%, reflecting generally positive views. Table 2 presents the distribution of participants’ self-reported attitudes, categorized into four levels: strong negative, negative, positive, and strong positive.

**Table 2.** Distribution of Participants by Levels of Attitudes Towards Autism (Weighted Sample).

Category	Frequency	Percent (%)
Strong Negative	2	0.1
Negative	222	9.3
Positive	1,326	55.8
Strong Positive	825	34.7
Total	2,375	100

As shown in Table 2, most respondents expressed favorable attitudes towards autistic individuals, with 55.8% reporting a positive attitude and 34.7% indicating a strong positive attitude. In total, 90.5% endorsed positive or strong positive attitudes, whereas only 9.5% reported negative or strong negative attitudes.

A chi-square goodness-of-fit test was conducted to examine whether attitudes were evenly distributed across the four categories (strong negative, negative, positive, and strong positive). The analysis revealed a significant deviation from a uniform distribution, χ^2^(3, *N* = 2,375) = 1,897.09, *p* < .001, Cramér's *V* = .52, indicating that responses were not evenly distributed but were instead heavily skewed towards the positive end of the scale.

The overall mean self-reported knowledge score was 64.66% (*SD* = 13.39), with a median of 66.67%, indicating a relatively high level of knowledge across age groups. For the analysis, respondents’ knowledge scores were divided into four quartiles (low, medium, high, and very high), as shown in Table 3.

**Table 3.** Distribution of Participants by Self-Reported Knowledge Level About Autism (Weighted Sample).

Category	Frequency	Percent
Low	0	0.0%
Medium	480	20.2%
High	1,333	56.1%
Very High	562	23.6%
Total	2,375	100.0%

As shown in Table 3, more than three-quarters of the participants (79.7%) reported a high or very high level of knowledge about autism, while 20.2% rated their knowledge as medium. A chi-square goodness-of-fit test indicated that knowledge levels differed significantly from a uniform distribution, χ^2^(3, *N* = 2,375) = 1,964.02, *p* < .001, Cramér's *V* = 0.53, suggesting that the majority of respondents demonstrated relatively high levels of knowledge about autism.

To further understand the factors shaping these findings, additional analyses were conducted to examine differences in attitudes and knowledge across key socio-demographic and variables (e.g., age, gender, education, income, ethnicity, religiosity, and familiarity with autism), as well as to evaluate predictors of attitudes towards autistic individuals.

### Age

**Figure 1.**
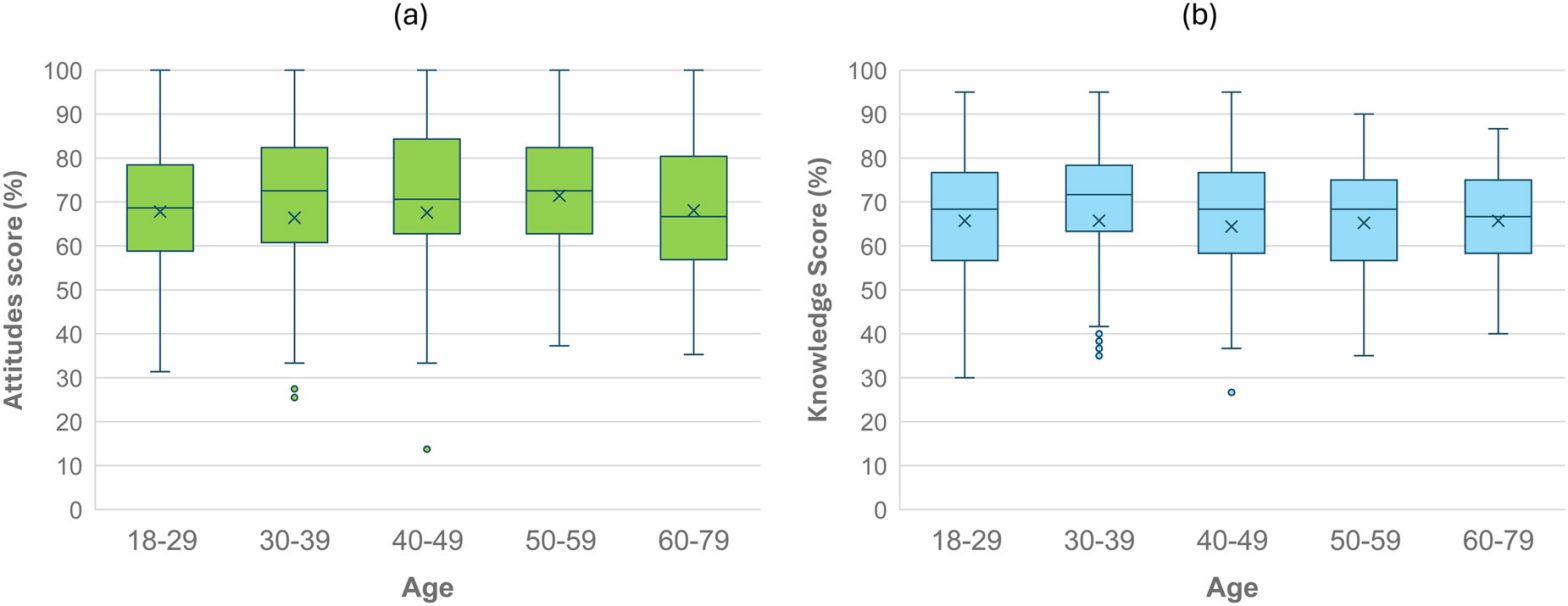
Figure 1ab presents box plots for attitudes (a) and knowledge (b) across the five age groups. The boxes represent the interquartile range (25th–75th percentiles), with the horizontal line inside each box indicating the median and the x indicating the average. The whiskers extend to the 10th and 90th percentiles, and dots denote outliers.

To explore whether attitudes and knowledge towards autistic individuals varied by age, participants were divided into five age groups for comparison. Figure 1a and b displays a box plot of the five age groups, highlighting the median scores and the overall distribution of *attitude* and Knowledge scores within each group.

As shown in Figure 1a, participants aged 50–59 reported the most favorable attitudes (median = 72.55%), followed closely by the 40–49 and 30–39 age groups (both medians = 70.59%). In contrast, the 18–29 age group reported less favorable attitudes (median = 68.63%), and the 60–79 group reported the lowest median score (median = 66.67%). A Kruskal–Wallis test revealed significant differences in attitudes between age groups, *H*(4) = 37.14, *p* < .001, Cohen's *d* = 0.24. Pairwise comparisons using Bonferroni correction indicated that both the 18–29 and 60–79 groups had significantly lower attitude scores than the 30–39, 40–49, and 50–59 groups (all *p* < .01). No statistically significant differences were found among the middle adult groups (30–39, 40–49, and 50–59), nor between the youngest (18–29) and oldest (60–79) groups. These findings suggest that attitudes towards autistic individuals are most positive during midlife (30s to 50s), and relatively less positive in early and later adulthood.

As shown in Figure 1b, participants aged 30–39 reported the highest knowledge scores (median = 69.87%), followed by those aged 40–49 (median = 68.33%). The 50–59 and 60–79 groups both exhibited median scores of 66.67%, while the lowest median score was observed among participants aged 18–29 (median = 65.00%). A Kruskal–Wallis test revealed significant differences in knowledge across age groups, *H*(4) = 61.68, *p* < .001, Cohen's *d* = 0.31. Pairwise comparisons with Bonferroni correction showed that the 30–39 group reported a significantly higher knowledge level than every other age group, including the 18–29, 40–49, 50–59, and 60–79 groups (*p* < .001 for all comparisons). Additionally, the 40–49 group demonstrated significantly higher knowledge scores than the 60–79 group (*p* = .024). No other age group comparisons reached statistical significance after adjustment. These findings suggest that self-reported knowledge about autism peaks significantly in individuals aged 30–39, remains relatively high in those aged 40–49, and is somewhat lower in early and later adulthood.

### Gender

**Figure 2.**
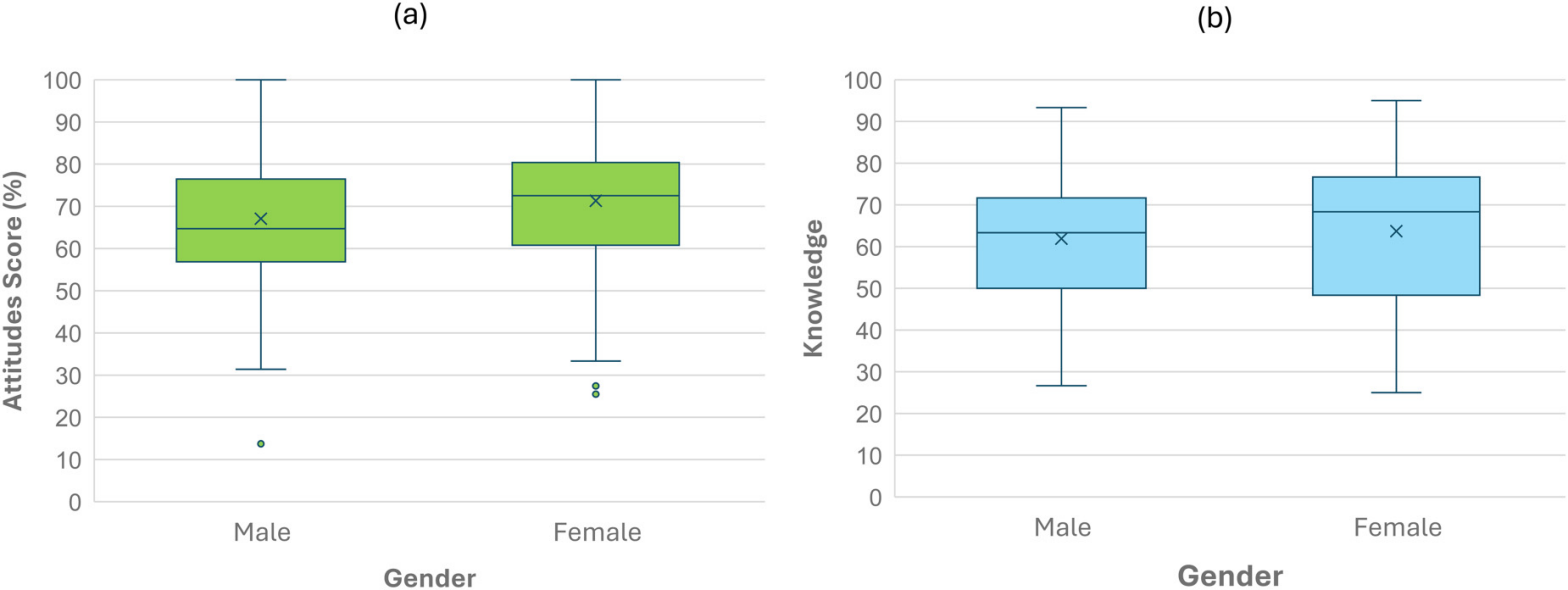
Figure 2ab presents box plots of male and female respondents for attitudes (a) and knowledge (b). The boxes represent the interquartile range (25th–75th percentiles), with the horizontal line inside each box indicating the median and the x indicating the average. The whiskers extend to the 10th and 90th percentiles, and dots denote outliers.

Participants’ attitudes and knowledge about autistic individuals were examined by gender, with the results presented in Figures 2a and b.

An analysis of attitudes towards autistic individuals revealed significant differences between male and female participants. On average, women reported more positive attitudes (*M* = 71.68%, *SD* = 13.31%) compared to men (*M* = 67.36%, *SD* = 14.12%). A Mann–Whitney *U* test confirmed that the gender difference was statistically significant, *U* = 631,671.50, *Z* = 10.07, *p* < .001, Cohen's *d* = 0.40. These findings suggest that women in the sample held significantly more favorable attitudes towards autistic individuals than men.

Gender differences were also observed in knowledge levels: Women reported slightly higher knowledge scores (*M* = 66.50%, *SD* = 13.98%) compared to men (*M* **=** 62.78%, *SD* = 12.47%), as shown in Figure 2b. A Mann–Whitney *U* test confirmed that this difference was statistically significant, *U* = 532,388.00, *Z* = 15.35, *p* < .001, Cohen's *d* = 0.63.

### Education

**Figure 3.**
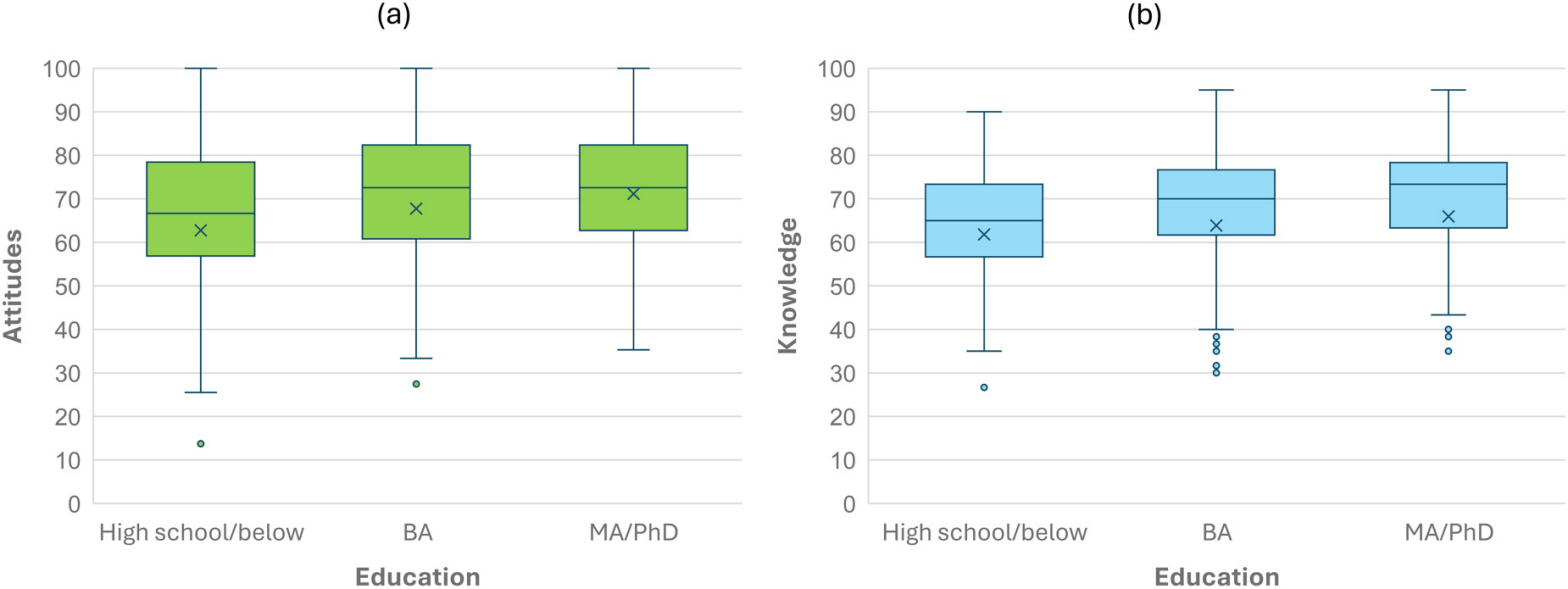
Figure 3ab presents box plots for attitudes (a) and knowledge (b) across the three levels of education. The boxes represent the interquartile range (25th–75th percentiles), with the horizontal line inside each box indicating the median and the x indicating the mean. The whiskers extend to the 10th and 90th percentiles, and dots denote outliers.

Participants’ attitudes and knowledge towards autistic individuals were examined by education level, with the results presented in Figure 3a and b.

Figure 3a presents the distribution of *attitudes* towards autistic individuals across education levels, highlighting clear differences in median scores among groups. A Kruskal–Wallis test revealed significant differences in attitudes towards autistic individuals across education levels, *H*(2) = 48.48, *p* < .001, Cohen's *d* = 0.28. Median attitude scores were 66.67% for participants with a high school education or below, and 70.59% for both those with a bachelor's degree and those with a master's or a doctoral degree. Pairwise comparisons with Bonferroni correction indicated that participants with a bachelor's degree reported significantly more positive attitudes than those with a high school education or below (*p* < .001), and participants with a master's or doctoral degree demonstrated significantly more positive attitudes than those with a high school education or below (*p* < .001). The difference between holders of bachelor's degrees and those with graduate degrees did not reach statistical significance (*p* = 1.000). These findings suggest that higher educational attainment is associated with more favorable attitudes towards autistic individuals.

Figure 3b presents the distribution of self-reported *knowledge* about autism across education levels, illustrating consistent increases in median scores with higher educational attainment.

A Kruskal–Wallis test revealed significant differences in knowledge scores across education groups, *H*(2) = 128.97, *p* < .001, Cohen's *d* = 0.46. Median knowledge scores were 63.33% for participants with a high-school education or below, 68.33% for those with a bachelor's degree, and 71.67% for those with a master's or doctoral degree. Pairwise comparisons using Bonferroni correction showed that all differences between the three groups were statistically significant: Holders of bachelor's degrees scored higher than those with a high school education or below (*p* < .001), and holders of graduate degrees scored higher than both the high school group (*p* < .001) and the bachelor's group (*p* < .021). These findings suggest a clear and consistent association between higher education and greater knowledge about autism.

### Income

**Figure 4.**
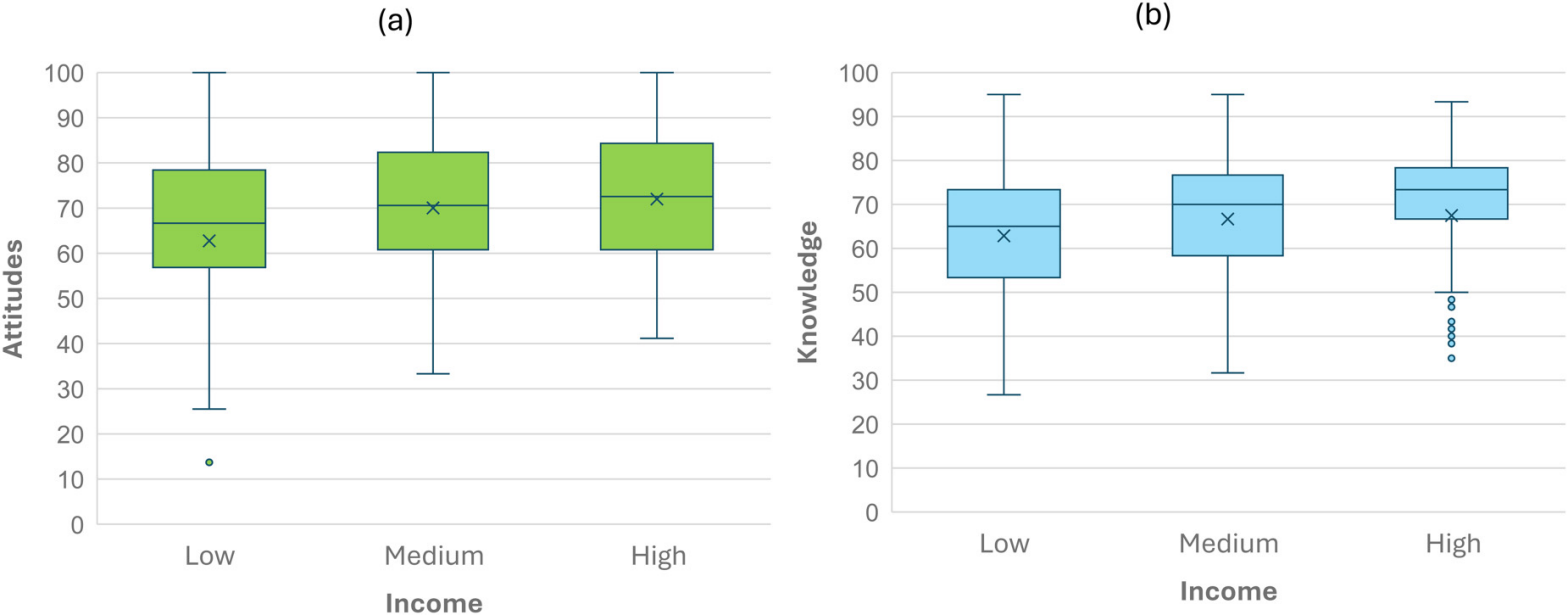
Figure 4ab presents box plots for attitudes (a) and knowledge (b) across the three levels of income. The boxes represent the interquartile range (25th–75th percentiles), with the horizontal line inside each box indicating the median and the x indicating the mean. The whiskers extend to the 10th and 90th percentiles, and dots denote outliers.

Participants’ attitudes towards and knowledge about autistic individuals were examined by income level, categorized as low (below 10,000 NIS), medium (10,001–20,000 NIS), and high (above 20,000 NIS), with the results presented in Figure 4a and b.

Median attitude scores increased from the low-income group to the medium- and high-income groups: Participants in the low-income group had a median score of 66.67%, while both the medium-income and high-income groups had a median of 70.59%. A Kruskal–Wallis test revealed significant differences in attitudes towards autistic individuals across income levels, *H*(2) = 51.62, *p* < .001, Cohen's *d* = 0.29. Pairwise comparisons with Bonferroni correction showed that both the medium- and high-income groups reported significantly more positive attitudes than the low-income group (*p* < .001 for both comparisons). The difference between the medium- and high-income groups did not reach statistical significance after adjustment (*p* = .782). These findings indicate that higher income is associated with more favorable attitudes towards autistic individuals, particularly when comparing the low-income group to those with medium or high incomes.

Figure 4b presents the distribution of knowledge scores across the three income groups, highlighting clear differences in median values. Median knowledge scores were 61.67% in the low-income group, 66.67% in the medium-income group, and 71.67% in the high-income group. Knowledge about autism varied significantly across income levels, as indicated by a Kruskal–Wallis test, *H*(2) = 125.40, *p* < .001. Cohen's *d* = 0.45. Pairwise comparisons with Bonferroni correction confirmed that each increase in income level was associated with significantly higher knowledge scores (*p* < .001 for all comparisons). These results demonstrate a strong and consistent relationship between higher income and greater self-reported knowledge about autism.

### Ethnicity

**Figure 5.**
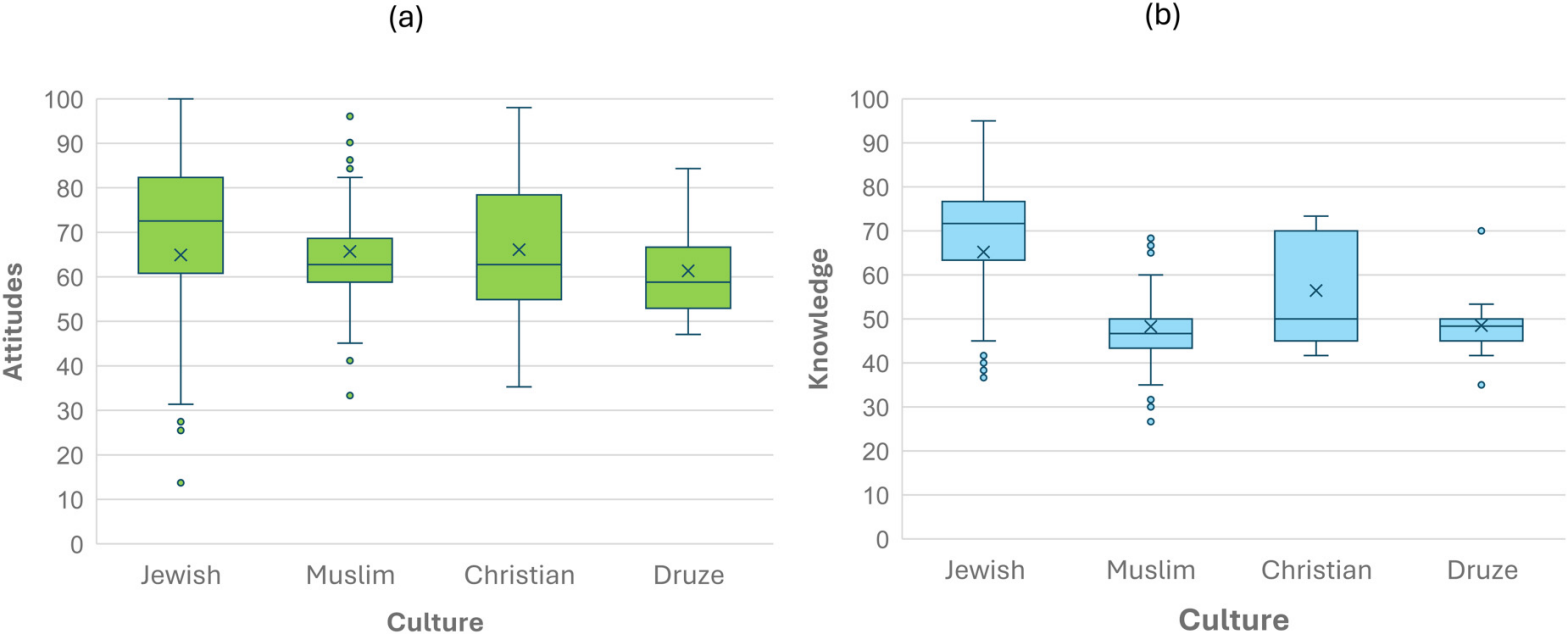
Figure 5ab presents box plots for attitudes (a) and knowledge (b) across different ethnicity groups. The boxes represent the interquartile range (25th–75th percentiles), with the horizontal line inside each box indicating the median and the x indicating the mean. The whiskers extend to the 10th and 90th percentiles, and dots denote outliers.

Participants’ attitudes and knowledge about autistic individuals were examined by ethnicity, with the results presented in Figure 5a and b. Figure 5a presents the distribution of self-reported *attitudes* towards autistic individuals across ethnic groups, highlighting notable differences in median scores.

Median attitude scores were highest among Jewish participants (70.59%), followed by Muslims (64.71%), Druze (62.28%), and Christians (60.84%). A Kruskal–Wallis test confirmed that these differences were statistically significant, *H*(3) = 112.92, *p* < .001, Cohen's *d* = 0.43. Pairwise comparisons with Bonferroni correction indicated significant differences in attitudes toward autism between Jewish participants and Muslim (*p* < .001), Druze (*p* < .001), and Christian (*p* = .009) participants, with Jewish participants reporting more positive attitudes overall. Differences among the non-Jewish groups did not reach statistical significance after adjustment. These findings suggest meaningful cultural variation in attitudes.

Figure 5b illustrates the distribution of self-reported knowledge about autism across cultural groups, revealing substantial disparities in median scores. Median knowledge scores were highest among Jewish participants (70.00%), followed by Christians (50.00%), and then both Druze and Muslims (both Medians = 46.67%). A Kruskal–Wallis test indicated that these differences were highly significant, *H*(3) = 821.94, *p* < .001, Cohen's *d* = 1.37. Pairwise comparisons with Bonferroni correction showed significant differences in autism-related knowledge across ethnic groups, with Jewish participants reporting greater knowledge about autism than Muslim, Druze, and Christian participants (*p* < .001 for all comparisons). Additionally, Christian participants reported significantly higher knowledge scores than Muslim participants (*p* = .004). The remaining differences among the non-Jewish groups (Druze vs. Muslim and Druze vs. Christian) were not statistically significant after correction. These findings suggest meaningful variation across ethnic groups in self-reported knowledge about autism.

### Religiosity

**Figure 6.**
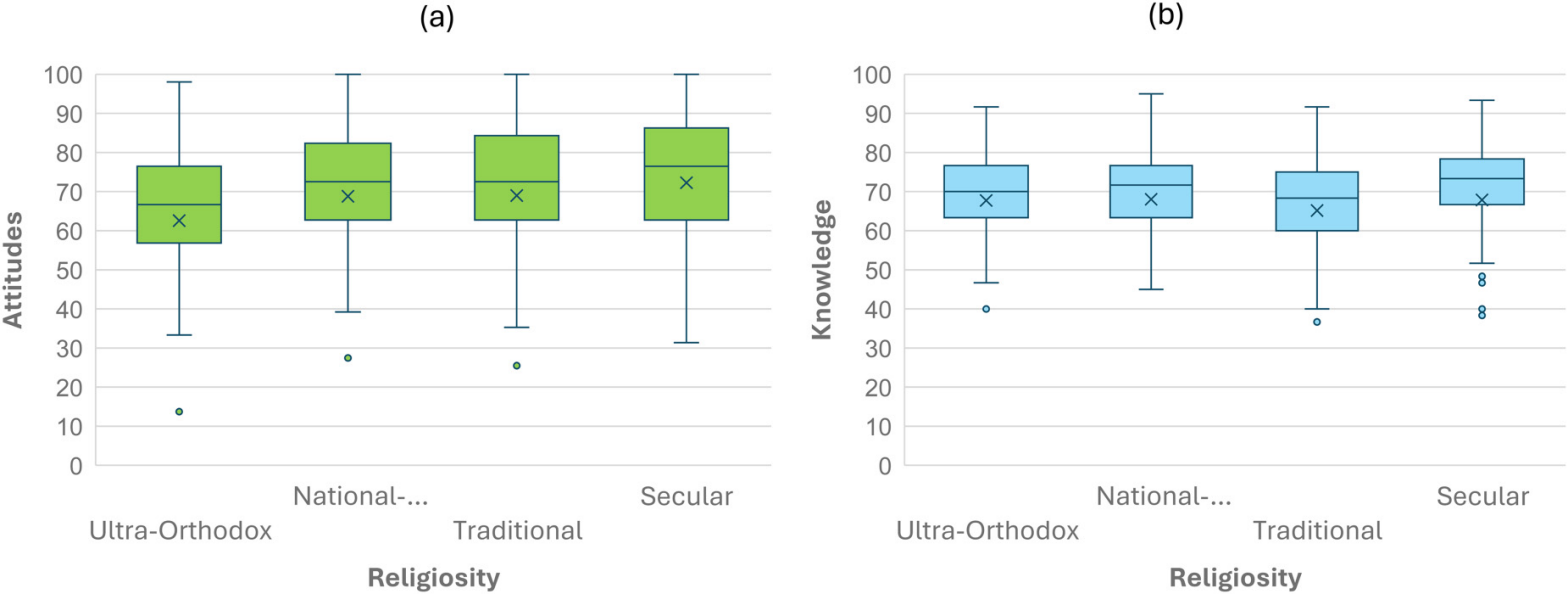
Figure 6ab presents box plots for attitudes (a) and knowledge (b) across different religiosity groups. The boxes represent the interquartile range (25th–75th percentiles), with the horizontal line inside each box indicating the median and the x indicating the mean. The whiskers extend to the 10th and 90th percentiles, and dots denote outliers

Participants’ attitudes and knowledge about autistic individuals were also examined by level of religiosity among Jews, categorized as Ultra-Orthodox, Religious Zionist, traditional, and secular. The results are presented in Figure 6a and b. Figure 6a displays the distribution of self-reported attitudes towards autistic individuals across the four groups, indicating distinct differences in median scores.

Median attitude scores were highest among secular participants (74.51%), followed by both traditional and Modern-Orthodox participants (both medians = 72.55%), and lowest among ultra-Orthodox participants (64.71%). A Kruskal–Wallis test revealed significant differences in attitudes towards autistic individuals across levels of religiosity, *H*(3) = 115.01, *p* < .001, Cohen's *d* = 0.47. Pairwise comparisons using Bonferroni correction showed that Ultra-Orthodox participants reported significantly less positive attitudes than all other groups, namely, Modern Orthodox (*p* < .001), traditional (*p* < .001), and secular (*p* < .001). Differences among the secular, traditional, and Modern-Orthodox groups did not reach statistical significance after adjustment. These findings indicate that while attitudes are generally positive and uniform across most segments of the Jewish population, the ultra-Orthodox group exhibited significantly less favorable attitudes.

Figure 6b presents the distribution of self-reported knowledge about autism across the different levels of religiosity. Median knowledge scores were highest among secular participants (73.33%), followed by Modern-Orthodox (70.00%), ultra-Orthodox (68.33%), and traditional participants (66.67%). A Kruskal–Wallis test confirmed significant differences in knowledge scores across groups defined by their level of religiosity, *H*(3) = 75.02, *p* < .001, Cohen's *d* = 0.37. Pairwise comparisons with Bonferroni correction revealed that secular participants reported significantly higher knowledge levels than traditional (*p* < .001), ultra-Orthodox (*p* < .001), and Modern-Orthodox (*p* = .002) participants. Additionally, Modern Orthodox participants scored significantly higher than traditional participants (*p* = .007). The ultra-Orthodox group did not differ significantly from either the traditional or Modern-Orthodox groups. These results suggest that secular individuals generally report the highest level of knowledge about autism, while differences among the other religious groups are more moderate.

### Knowing a Person With Autism

**Figure 7.**
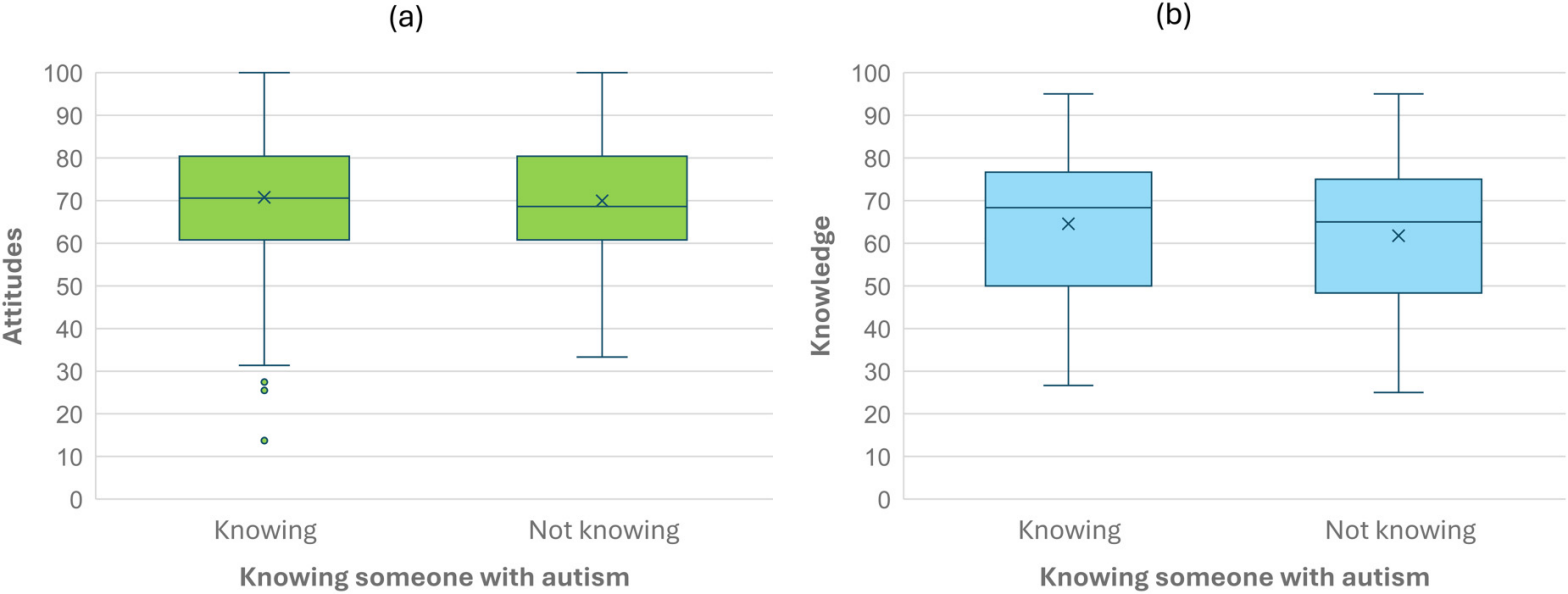
Figure 7ab presents box plots for attitudes (a) and knowledge (b) based on whether respondents personally knew someone on the autism spectrum. The boxes represent the interquartile range (25th–75th percentiles), with the horizontal line inside each box indicating the median and the x indicating the mean. The whiskers extend to the 10th and 90th percentiles, and dots denote outliers.

Participants’ attitudes towards and knowledge about autistic individuals were examined based on whether they personally knew someone on the autism spectrum. The results are presented in Figure 7a and b. Figure 7a displays the differences in *attitudes* towards autistic individuals based on whether participants personally knew someone on the autism spectrum.

The results indicated that participants who reported knowing someone with autism expressed significantly more positive attitudes (median = 70.59%) than those who did not know a person with autism (median = 68.63%), *U* = 757,850.50, *Z* = 2.42, *p* = .016, Cohen's *d* = 0.08. Figure 7b displays the differences in knowledge about autistic individuals based on whether or not participants personally knew someone on the autism spectrum.

Participants who knew someone with autism had significantly higher knowledge scores (median = 68.33%) compared to those who did not (median = 65.00%), *U* = 704,203.50, *Z* = 5.34, *p* < .001, Cohen's *d* = 0.187. These findings suggest that personal familiarity with autism is associated with both more favorable attitudes and greater self-reported knowledge.

### Regression Analysis

A stepwise multiple regression analysis was conducted to assess the extent to which knowledge about autism and demographic variables predicted attitudes towards autistic individuals. To avoid a reduction in sample size, religiosity was excluded from this analysis, as it was reported only by Jewish participants. The results of the final model are presented in Table 4.

**Table 4.** Summary of Stepwise Multiple Regression Analysis for Variables Predicting Attitudes Towards Autistic Individuals in the Weighted Sample (Final Model).

Variable	B	SE B	β	t	*p*
(Constant)	47.22	1.39		34.01	<.001
Knowledge about autism	0.24	0.02	.23	11.18	<.001
Gender	3.47	0.56	.13	6.15	<.001
Income	1.25	0.23	.12	5.52	<.001
Education	0.86	0.31	.06	2.77	.006

*Note.* The variables are presented in the order they entered the stepwise regression model. Total adjusted R^2^ for the final model = .115 (F(4, 2324) = 76.92, *p* < .001). Age, ethnicity (sector), and knowing someone with autism were excluded from the final model as they were not significant predictors.

The stepwise analysis yielded four significant models. In Model 1, knowledge about autism emerged as the strongest single predictor of positive attitudes, R^2^ = .082, F(1, 2327) = 208.86, *p* < .001. Subsequent steps significantly added demographic variables: income in Model 2 (ΔR^2^ = .014, *p* < .001), gender in Model 3 (ΔR^2^ = .018, *p* < .001), and education in Model 4 (ΔR^2^ = .003, *p* = .006).

The final model (Model 4) accounted for 11.7% of the total variance in attitudes towards autistic individuals, F(4, 2324) = 76.92, *p* < .001. In this model, knowledge remained the most robust predictor (B = 0.237, β = .229, *p* < .001), meaning that higher knowledge is associated with more positive attitudes. Gender (β = .125, *p* < .001), income (β = .121, *p* < .001), and education (β = .059, *p* = .006) also contributed significantly to the model. Variables such as age, ethnicity, and knowing someone with autism did not emerge as significant predictors and were thus excluded from the final model. These findings confirm that while demographic factors like gender and income play a role, knowledge about autism is the primary and strongest predictor of positive attitudes.

## Discussion

This study examined attitudes and knowledge about autism in Israeli society. By analyzing perceptions across diverse demographic groups, it sought to identify gaps in understanding that may contribute to stigma, misconceptions, and the social exclusion of autistic individuals. Given Israel's cultural, religious, and socioeconomic diversity, the findings are crucial for developing culturally sensitive educational and support strategies that promote inclusion and awareness. Moreover, this research addresses a gap in the international literature by providing cross-cultural data on how demographic and cultural contexts shape public attitudes towards autism, thereby informing targeted interventions worldwide.

The results revealed several key findings. First, attitudes towards autism were predominantly positive. Second, the level of knowledge about autism was generally high. Third, knowledge levels and attitudes were significantly correlated. In addition, demographic and social factors—such as gender, age, education, income, and ethnicity—were found to influence both attitudes and knowledge. Finally, personal experience with an autistic individual was associated with more positive attitudes. The following sections discuss each of these findings in relation to previous research and relevant theoretical frameworks.

### Attitudes and Knowledge Levels

Most respondents reported positive (55.08%) or very positive (34.70%) attitudes towards autistic individuals, indicating that approximately 90.75% of participants expressed generally favorable views. These findings align with previous international studies (e.g., Kuzminski et al., 2019) and reflect the integrative approach to disability, which emphasizes social integration and equality (Hastings et al., 1998). However, as noted in earlier research, self-reported measures may be influenced by a social desirability bias (Dickter et al., 2020; Wolff et al., 1996). Thus, although the current results suggest a generally supportive public perception, the presence of a small subgroup (9.50%) expressing negative views underscores the ongoing need for public education and stigma-reduction initiatives.

Similarly, a substantial majority of respondents (79.7%) reported high or very high levels of knowledge about autism (46% high; 25% very high), suggesting that public awareness is relatively well developed. However, approximately 20.2% of participants reported only moderate or low levels of knowledge, indicating that important gaps in understanding still remain. These findings highlight the need for continued educational outreach, particularly among underrepresented or socioeconomically disadvantaged groups, to ensure more equitable access to accurate and comprehensive information about autism. While both attitudes and knowledge levels appear relatively high within Israeli society, it is essential to identify the factors that shape them. The following sections discuss the influence of demographic, social, and experiential variables on knowledge levels and attitudes towards autism.

### Age

Previous research has produced mixed findings regarding the relationship between age, attitudes, and knowledge about autism. Some studies report no significant association between age and attitudes (Kuzminski et al., 2019; Ryan, 2013), while others suggest that attitudes become more positive with increasing age (Aubé et al., 2021). More recent work, however, indicates that age is associated with both attitudes and knowledge, although the direction of this relationship is not always consistent (Zarokanellou et al., 2025). These discrepancies highlight the complexity of age-related effects and underscore the need for a more nuanced understanding of how different stages of adulthood relate to perceptions of autism.

The present findings reveal a non-linear pattern in both attitudes and knowledge across age groups. Attitudes towards autistic individuals were most positive among adults aged 30–59, with both younger adults (18–29) and older adults (60–79) reporting significantly less favorable attitudes. Notably, no differences emerged among the adults aged 30–59, suggesting a relatively stable period of more positive attitudes in midlife. In contrast, knowledge about autism peaked more specifically in early mid-adulthood, with individuals aged 30–39 reporting significantly higher knowledge than all other age groups. Knowledge remained relatively high in the 40s but declined somewhat in both younger and older groups.

Importantly, the patterns for attitudes and knowledge only partially overlapped. While knowledge was highest in the 30s, the most favorable attitudes extended across a broader midlife range (from 30s to 50s). This divergence suggests that although knowledge contributes to attitudes, it does not fully account for them. Other factors characteristic of midlife—such as increased life experience, social roles, or interpersonal exposure—may play an additional role in shaping more positive perceptions.

The relatively less favorable attitudes observed among older adults may reflect generational differences. Individuals in older cohorts grew up during periods when autism was less visible, often misunderstood, and framed primarily in clinical or deficit-based terms. Limited exposure and outdated representations may contribute to less favorable attitudes. At the same time, the finding that younger adults reported both lower levels of knowledge and less positive attitudes than adults aged 30–59 is noteworthy. Although younger generations are often assumed to be more accepting, their knowledge may be shaped by informal and sometimes unreliable sources, such as social media, which can present simplified or inconsistent portrayals of autism (Jones et al., 2021; O’Reilly et al., 2022).

Another plausible explanation for the midlife advantage is the increased likelihood of direct or indirect contact with autism through parenting, educational settings, or professional contexts. Such experiences may foster both greater understanding and more nuanced attitudes. In contrast, younger individuals may endorse general inclusivity but lack the experiential grounding that supports deeper knowledge and consistently positive attitudes.

Overall, these findings underscore the importance of considering age as a complex, non-linear factor in autism-related perceptions. The results highlight the need for targeted educational efforts, particularly among younger adults, to promote accurate knowledge and foster more positive attitudes early in adulthood, while also addressing potential gaps in older populations through accessible, evidence-based outreach.

### Gender

The findings of the present study suggest that gender plays a meaningful role in shaping attitudes towards autistic individuals. Women exhibited significantly more positive attitudes towards individuals with autism than men. Similarly, women reported higher levels of knowledge about autism compared to men. These findings are consistent with some previous studies, according to which women generally demonstrate more positive attitudes and greater knowledge about autism compared to men (Durmaz et al., 2024; Kuzminski et al., 2019). This finding is also supported by other studies revealing that gender may influence attitudes towards challenged populations (Babik & Gardner, 2021). This may reflect differences in socialization, empathy-related traits, or experiences with individuals on the spectrum. While the statistically significant differences between genders suggest that males could be a specific target for interventions addressing knowledge of and attitudes towards autism, the widespread attitudes and knowledge within both groups complicates this approach. Therefore, to maximize impact, interventions aimed at improving attitudes towards autism and increasing knowledge should target both males and females.

### Education

The current findings clearly indicate that higher levels of education are associated with more positive attitudes towards autism. Respondents with academic degrees, both bachelor's and postgraduate degrees, reported more favorable attitudes towards autistic individuals than those with a high school education or less. Although participants with master's or doctoral degrees demonstrated slightly more positive attitudes than holders of bachelor's degrees, this difference was not statistically significant.

These results are consistent with prior studies conducted across diverse cultures, which have shown that higher educational attainment predicts more accepting attitudes towards autistic individuals and stronger support for inclusive education (Cage et al., 2018; Dillenburger et al., 2013; Kouznetsov & Jelastopulu, 2023; Surmen et al., 2015). This association may reflect the fact that individuals with higher education are more likely to hold an evidence-based understanding of disabilities, thereby challenging stereotypes and misconceptions (Ryan, 2013). Moreover, university environments often promote ethical and inclusive values, thus reinforcing respect for human diversity (Jarman et al., 2023; Svendby, 2024). In contrast, limited educational opportunities may restrict access to accurate information, fostering misinformation and negative perceptions (Nissim-Kanety et al., 2006; Wang et al., 2021). Individuals with lower levels of education may rely more heavily on inaccurate or sensationalized media portrayals, which can reinforce stigma and social distance towards disability (Nissim-Kanety et al., 2006).

The present study also revealed a clear linkage between education level and knowledge about autism. Compared to participants with only a high school education or less, those participants with academic degrees reported a greater understanding of the characteristics of autism, its diagnosis, and the support needs of individuals with autism. Furthermore, individuals with master's or doctoral degrees had higher knowledge scores than those with bachelor's degrees, suggesting that advanced academic education is linked to a deeper and more nuanced understanding of autism. These findings align with previous research across various cultural and professional contexts (Surmen et al., 2015).

### Income

The findings of the present study indicate that individuals with middle- and high-income levels tend to hold more positive attitudes towards autism than those with low income. However, no significant differences were found between the middle- and high-income groups, suggesting that the assumption that high income directly predicts more positive attitudes towards autism was only partially supported.

This pattern aligns with earlier studies (Babik & Gardner, 2021; Kuzminski et al., 2019), which suggest that capitalist social structures emphasizing independence and productivity may link low income to diminished social status. Such perceptions can, in turn, foster a tendency among low-status individuals to devalue marginalized populations, including persons with disabilities. Similarly, Nissim-Kanety et al. (2006) proposed psychosociological explanations for the prevalence of negative attitudes among low-income individuals, arguing that these attitudes may stem from a perceived socioeconomic threat. The absence of significant differences between middle- and high-income participants may reflect their comparable self-perceived social status, as middle-income individuals often identify more closely with high-income groups, leading to reduced bias towards marginalized populations.

Beyond attitudinal differences, the present study found a consistent relationship between income level and levels of knowledge about autism; that is, each increase in income was associated with significantly higher knowledge scores. These findings correspond with prior research indicating that income is positively related to knowledge about autism (Al-Juhaishi et al., 2025; Al-Ruwaili et al., 2024; Kaman et al., 2023; Younes et al., 2025). However, several of these studies have demonstrated that the effect of income is largely mediated by education (Al-Juhaishi et al., 2025; Al-Ruwaili et al., 2024; Kaman et al., 2023; Younes et al., 2025), suggesting that educational access may be the key factor linking socioeconomic resources to knowledge and attitudes towards autism.

### Culture and Ethnicity

The present study identified significant variations across ethnic groups in both attitudes and knowledge about autism. Although all groups generally expressed positive views, Jewish participants reported more positive attitudes toward autism and greater autism-related knowledge compared to Arab and Druze participants. These differences suggest that cultural and structural factors jointly shape how autism is perceived and discussed within Israel's multicultural society. The findings reflect Israel's complex sociocultural mosaic, in which Jewish, Arab, and Druze communities differ in language, education, access to healthcare, and prevailing social norms regarding disability and neurodiversity.

Cultural frameworks influence how developmental differences are interpreted within families and communities. Prior research in Israel has shown that cultural and religious traditions shape parental beliefs, coping strategies, and patterns of help-seeking when facing developmental challenges (Kandel et al., 2004; Weinberg & Sebian, 1980). Exposure to biomedical and developmental perspectives on autism may differ across communities due to variation in educational access, healthcare engagement, and public discourse. In some Arab and Druze communities, developmental disorders may be more likely to be understood through familial or spiritual frameworks, which may contribute to delayed identification and reduced public discussion of neurodiversity. These contextual factors may also contribute to uncertainty, stigma, or reluctance to seek formal assessment and intervention services.

At the same time, differences in attitudes and knowledge may also reflect broader socioeconomic and educational disparities rather than purely cultural distinctions. According to national demographic data (Central Bureau of Statistics, 2023), average levels of education and income differ across Jewish, Arab, and Druze populations in Israel. These structural differences may influence exposure to inclusive educational settings, public awareness campaigns, and professional training opportunities related to developmental and neurodiverse conditions. Building on cross-cultural evidence that education, socioeconomic resources, and cultural beliefs interact to shape public perceptions of autism (Gemegah et al., 2021; Jorm, 2000; Kuzminski et al., 2019), the current findings likely reflect the combined influence of these contextual determinants.

Future research should aim to develop culturally adapted interventions that promote awareness, acceptance, and early identification across all segments of Israeli society. Partnerships among schools, healthcare systems, religious leaders, and community organizations can help ensure that information about autism is disseminated in linguistically and culturally appropriate ways. Such collaborative efforts are essential for reducing stigma, increasing equity in access to services, and supporting the inclusion of autistic individuals across Israel's diverse cultural landscape.

### Religiosity

The present findings highlight religiosity as a meaningful socio-cultural factor associated with variability in both attitudes towards autistic individuals and self-reported knowledge about autism. While overall attitudes were generally positive across groups, significant differences emerged across levels of religiosity, with ultra-Orthodox participants reporting less positive attitudes toward autism compared to the other groups. These differences suggest that cultural context, community norms, and varying levels of exposure to neurodevelopmental conditions may shape social perceptions (e.g., Grinker, 2020). Secular participants also reported relatively higher levels of autism-related knowledge and positive attitudes, with knowledge differences showing a clearer gradient across groups than attitudes. This pattern may reflect differential access/exposure to information, educational resources, and inclusion practices, which are often more prevalent in less insular communities (Mavropoulou & Sideridis, 2014). Importantly, the relative similarity in attitudes among secular, traditional, and Modern Orthodox participants, despite differences in knowledge, suggests that positive orientations towards autistic individuals may not depend solely on formal knowledge, but may also be affected by broader societal values and exposure. Together, these findings underscore the need for culturally sensitive outreach and education initiatives, particularly within communities with more limited exposure to autism-related information and services, to promote both understanding and acceptance of autism.

### Knowing a Person With Autism

Consistent with principles of Contact Theory (Allport, 1954), participants who reported knowing an autistic individual also reported greater autism-related knowledge and more positive attitudes toward autism compared to those who did not. This finding supports previous research suggesting that familiarity with autistic individuals may promote empathy and reduce stigma (Morris et al., 2004; Nevill & White, 2011).

However, it is important to note that the present study did not assess the depth, frequency, or quality of contact with the autistic person. As such, the results only capture the presence or absence of acquaintance, without differentiating between close family relationships, friendships, or indirect familiarity (e.g., knowing a student, colleague, or neighbor). The observed differences, while statistically significant, were modest in magnitude, indicating that mere acquaintance may not be sufficient to produce substantial changes in attitudes or knowledge. Prior work has shown that the quality and context of interaction, rather than mere exposure, play a critical role in shaping meaningful understanding and acceptance (Campbell & Barger, 2011).

In the Israeli context, where opportunities for inclusive education and social integration differ across regions and cultural groups, these findings highlight the importance of promoting sustained, positive contact experiences between autistic and non-autistic individuals. Structured opportunities for collaboration and shared goals, such as inclusive education programs, workplace initiatives, and community projects, may strengthen the impact of social familiarity and contribute to reducing stigma. Future studies should therefore distinguish between different types of contact and examine how the closeness and quality of these relationships influence both knowledge and attitudes towards autism.

### Factors Influencing Attitudes Towards Autism

Beyond the examination of individual demographic factors, a stepwise multiple regression analysis was conducted to determine the relative contribution of knowledge about autism and demographic variables in predicting attitudes towards autistic individuals. Religiosity was not included in this analysis, as it was assessed only among Jewish participants, and its inclusion would have substantially reduced the sample size. The findings revealed that knowledge about autism was the most powerful and consistent predictor of attitudes, even after accounting for demographic variables such as gender, income, and education. This result underscores the central role of an informed understanding of autism in shaping how individuals perceive and relate to autistic people.

Although demographic characteristics contributed modestly to the prediction of attitudes, the dominant influence of knowledge suggests that it plays an independent and cross-cutting role in shaping public perceptions. The stepwise analysis demonstrated that knowledge alone accounted for a meaningful proportion of variance in attitudes (Model 1), with additional, but smaller, contributions from income, gender, and education in subsequent models. In the final model, knowledge remained the strongest predictor, while gender, income, and education also contributed significantly, albeit to a lesser extent. These findings are consistent with previous research showing that greater autism-related knowledge is associated with lower stigma, more positive attitudes toward autism, and more inclusive behaviors (Gillespie-Lynch et al., 2015; Kuzminski et al., 2019).

Importantly, variables such as age, ethnicity, and personal familiarity with autism did not emerge as significant predictors, suggesting that knowledge may be more influential than direct contact or broader sociodemographic background in shaping attitudes. Overall, the pattern observed here highlights the unique and robust contribution of knowledge to attitudes towards autistic individuals, emphasizing its broad relevance across gender, socioeconomic, and educational groups, and reinforcing the importance of educational and awareness-based interventions.

### Limitations and Future Research

Several limitations of the present study should be acknowledged. First, the reliance on self-report questionnaires may have introduced social desirability or response biases, potentially leading participants to overstate positive attitudes or knowledge. Second, familiarity with autism was measured dichotomously (“knowing a person with autism”), without accounting for the depth, frequency, or quality of contact—factors that could meaningfully moderate its effect on attitudes and knowledge levels.

Moreover, the regression model explained approximately 11.7% of the total variance in attitudes, indicating that while knowledge plays a central role, attitudes towards autism are shaped by a broader constellation of unmeasured factors. Emotional, cultural, and interpersonal influences, such as empathy, social norms, and personal values, likely contribute significantly to public perceptions of autism beyond the demographic and cognitive variables examined here.

Future research should employ longitudinal and mixed-method (quantitative and qualitative) designs to trace how attitudes evolve over time and to capture the lived experiences that shape them. Expanding future models to include affective, social, and cultural dimensions—such as empathy, perceived similarity, and community-level values—may offer a more comprehensive understanding of the mechanisms underlying acceptance and understanding of autism within diverse populations.

Although post-stratification weights were applied, the sample was recruited primarily through convenience-based methods and therefore may not fully represent the broader Israeli population. In addition, the survey was administered only in Hebrew and Arabic, which may have limited participation among other linguistic groups, including Russian speakers. The study also did not capture finer distinctions within the Jewish sample, such as ethnic origin or immigration background.

### Conclusions and Implications

This study provides a broad view of how autism is understood within Israel's multicultural society. The findings indicate that the level of knowledge and demographic background, including income, gender, and education, jointly shape public attitudes towards autism. Among these factors, knowledge consistently emerged as the strongest predictor, suggesting that understanding and awareness are central to acceptance.

These results emphasize the need for knowledge-based, culturally responsive initiatives that address both informational and social dimensions of stigma reduction. Expanding autism education within schools, professional training, and public media, particularly in underrepresented communities, may reduce misconceptions and promote inclusion. To enhance the impact of autism awareness initiatives, educational interventions should target populations with a lower level of knowledge and less positive attitudes—such as younger and older adults, men, and individuals with lower SES—and be culturally adapted to diverse societies. Equally important is the creation of sustained, meaningful contact between autistic and non-autistic individuals in educational, occupational, and community settings, fostering empathy and social cohesion.

## Appendix A

### Modifications to the Societal Attitudes Towards Autism (SATA) Questionnaire

**Table:**

**Question Number in the Current Study**	**Original Question in the Australian Article**	**Modification Made in the Current Study**
18	Movies such as “Rainman” show an accurate depiction of all autistic people	"Do movies or series in cinema, television, or online present an accurate picture of all autistic people?"
3	People with Autism should have the opportunity to go to university	"Should people with autism have the opportunity to study in academia?"
4	People with Autism should not have children	People with autism have the right to have children.
12	People with Autism should be encouraged to marry a person with Autism	People with autism should be encouraged to marry people with autism/special needs.
16	I would be uncomfortable hugging a person with Autism	I will be comfortable hugging someone with Autism.

Additional Elements:

- Added Question:

Question no. 15: Would you put your child in a classroom with children with autism?

- Open-Ended Question:

Do you have an additional insight/comment that was not expressed in the questionnaire and that you think should have been addressed?

- Omitted Question:

The character “Sheldon” in Big Bang Theory is an accurate depiction of all autistic people (omitted due to lack of familiarity within the Israeli population).

- Questionnaire Link:

The questionnaire was adapted to an electronic version: a collaborative form via the Google Forms platform. Here is a link to the questionnaire: https://docs.google.com/forms/d/e/1FAIpQLSd8NDudt6taGZWVf6rEZtMshf1JZv2G4bQQ5UzWogDcZNlcrA/viewform

## Appendix B

### SATA (Societal Attitudes Towards Autism) Questionnaire

The questionnaire is anonymous and takes less than 10 minutes to complete.

It is written in the masculine form for convenience only but is intended for both men and women.

Thank you very much for your cooperation!

#### Socio-Demographic Information

Gender: Male/Female

Religion: Jewish/Muslim/Christian/Other

Religious affiliation: Ultra-Orthodox/Modern Orthodox (National Religious)/Traditional/Secular/

Age: 18–29/30–39/40–49/50–59/60–69/70–79/80+

Occupation: __________________________

Education: High school graduate (Yes/No), Bachelor's (Yes/No), Master's (Yes/No), Doctorate (Yes/No)

Place of residence: __________________________

Monthly household income: Low/Medium/High

Do you know and/or have you spent some time with a person with autism?

[ ] Yes
[ ] No

#### Social Attitudes Towards People With Autism

Please select one answer for each question:

1 = Strongly agree | 2 = Agree | 3 = Disagree | 4 = Strongly disagree

1. People with autism should not be in romantic relationships.
  [ ] Strongly agree
  [ ] Agree
  [ ] Disagree
  [ ] Strongly disagree
2. People with autism should have the opportunity to study in institutions of higher education.
  [ ] Strongly agree
  [ ] Agree
  [ ] Disagree
  [ ] Strongly disagree
3. People with autism have the right to have children.
  [ ] Strongly agree
  [ ] Agree
  [ ] Disagree
  [ ] Strongly disagree
4. People with autism should be placed in an institution for their own safety and the safety of others.
  [ ] Strongly agree
  [ ] Agree
  [ ] Disagree
  [ ] Strongly disagree
5. If a treatment center for people with autism opened near my home, I would consider moving.
  [ ] Strongly agree
  [ ] Agree
  [ ] Disagree
  [ ] Strongly disagree
6. People with autism cannot live independently.
  [ ] Strongly agree
  [ ] Agree
  [ ] Disagree
  [ ] Strongly disagree
7. I would be afraid to be around a person with autism.
  [ ] Strongly agree
  [ ] Agree
  [ ] Disagree
  [ ] Strongly disagree
8. A person with autism is an emotional burden on their family.
  [ ] Strongly agree
  [ ] Agree
  [ ] Disagree
  [ ] Strongly disagree
9. I would feel comfortable sitting next to a person with autism in class.
  [ ] Strongly agree
  [ ] Agree
  [ ] Disagree
  [ ] Strongly disagree
10. A person with autism is a financial burden on their family.
  [ ] Strongly agree
  [ ] Agree
  [ ] Disagree
  [ ] Strongly disagree
11. People with autism should be encouraged to marry other people with autism/special needs.
  [ ] Strongly agree
  [ ] Agree
  [ ] Disagree
  [ ] Strongly disagree
12. People with autism cannot form relationships or express emotions.
  [ ] Strongly agree
  [ ] Agree
  [ ] Disagree
  [ ] Strongly disagree
13. Children with autism should be fully included in regular classrooms.
  [ ] Strongly agree
  [ ] Agree
  [ ] Disagree
  [ ] Strongly disagree
14. Would you enroll your child in a class with children with autism?
  [ ] Strongly agree
  [ ] Agree
  [ ] Disagree
  [ ] Strongly disagree
15. I would feel comfortable hugging a person with autism.
  [ ] Strongly agree
  [ ] Agree
  [ ] Disagree
  [ ] Strongly disagree
16. People with autism cannot understand the emotions of others.
  [ ] Strongly agree
  [ ] Agree
  [ ] Disagree
  [ ] Strongly disagree

#### Societal Views and Ideas

17. Movies or TV shows portray an accurate picture of all autistic people.
  [ ] Strongly agree
  [ ] Agree
  [ ] Disagree
  [ ] Strongly disagree
18. Autistic people remain childlike forever.
  [ ] Strongly agree
  [ ] Agree
  [ ] Disagree
  [ ] Strongly disagree
19. Autism is presented in the news in a balanced way.
  [ ] Strongly agree
  [ ] Agree
  [ ] Disagree
  [ ] Strongly disagree
20. One can tell if a person is autistic just by looking at them.
  [ ] Strongly agree
  [ ] Agree
  [ ] Disagree
  [ ] Strongly disagree
21. Vaccines do not cause autism.
  [ ] Strongly agree
  [ ] Agree
  [ ] Disagree
  [ ] Strongly disagree
22. Autistic people can choose which behaviors to display in public.
  [ ] Strongly agree
  [ ] Agree
  [ ] Disagree
  [ ] Strongly disagree
23. Autistic people experience emotions differently than non-autistic people.
  [ ] Strongly agree
  [ ] Agree
  [ ] Disagree
  [ ] Strongly disagree
24. Autistic people do not understand sarcasm or humor and take them literally.
  [ ] Strongly agree
  [ ] Agree
  [ ] Disagree
  [ ] Strongly disagree
25. Autistic people can learn to become less sensitive to stimuli such as noise, touch, or smell.
  [ ] Strongly agree
  [ ] Agree
  [ ] Disagree
  [ ] Strongly disagree
26. Autistic people struggle to know what to do in social situations.
  [ ] Strongly agree
  [ ] Agree
  [ ] Disagree
  [ ] Strongly disagree
27. Autism is a permanent condition that does not change from day to day.
  [ ] Strongly agree
  [ ] Agree
  [ ] Disagree
  [ ] Strongly disagree

#### Characteristics of Autism

28. Autism never appears in adults.
  [ ] Strongly agree
  [ ] Agree
  [ ] Disagree
  [ ] Strongly disagree
29. Autistic people are sensitive to certain stimuli such as noise, touch, and smell.
  [ ] Strongly agree
  [ ] Agree
  [ ] Disagree
  [ ] Strongly disagree
30. All autistic people have intellectual disabilities.
  [ ] Strongly agree
  [ ] Agree
  [ ] Disagree
  [ ] Strongly disagree
31. Autism includes a range of symptoms and varying levels of severity.
  [ ] Strongly agree
  [ ] Agree
  [ ] Disagree
  [ ] Strongly disagree
32. Autism exists only in children.
  [ ] Strongly agree
  [ ] Agree
  [ ] Disagree
  [ ] Strongly disagree
33. All autistic people demonstrate genius-level intellect.
  [ ] Strongly agree
  [ ] Agree
  [ ] Disagree
  [ ] Strongly disagree

#### Advantages and Challenges of Autism

34. All autistic people share the same strengths.
  [ ] Strongly agree
  [ ] Agree
  [ ] Disagree
  [ ] Strongly disagree
35. All autistic people share the same challenges.
  [ ] Strongly agree
  [ ] Agree
  [ ] Disagree
  [ ] Strongly disagree
36. The level of support required by autistic people changes with age.
  [ ] Strongly agree
  [ ] Agree
  [ ] Disagree
  [ ] Strongly disagree
37. Autistic people can be employed in jobs with standard wages.
  [ ] Strongly agree
  [ ] Agree
  [ ] Disagree
  [ ] Strongly disagree

Do you have any additional comments that you believe should be addressed?

____________________
